# Impact of Calcium Lactate Pretreatment on Enamel Fluoride Uptake: A Comparative In Vitro Study of Different Fluoride Types and Concentrations

**DOI:** 10.3390/jfb15090269

**Published:** 2024-09-16

**Authors:** Fjolla Kullashi Spahija, Ivana Sutej, Kresimir Basic, Kreshnik Spahija, Kristina Peros

**Affiliations:** 1 Department of Pharmacology, School of Dental Medicine, 10000 Zagreb, Croatia; isutej@sfzg.hr (I.S.); basic@sfzg.hr (K.B.); 2 Dental Policlinic Center, 30000 Peja, Kosovo; kreshnikspahija@hotmail.com

**Keywords:** calcium lactate, sodium fluoride, monofluorophosphate, aminofluoride

## Abstract

(1) Background: This study aimed to establish the effect of calcium lactate enamel pretreatment related to different fluoride types and concentrations on the enamel uptake of alkali-soluble fluorides. (2) Materials: In a blind and randomized in vitro study, a total of 60 teeth are used. The first 30 teeth were cut and randomly allocated into one of the following treatments: (A) calcium lactate pretreatment followed by three different fluoride solutions; (B) the “Fluoride only” group, with slabs treated with three different fluoride solutions; (C) the “Calcium only” group, with slabs treated with calcium lactate solution; (D) slabs treated with deionized water (negative control group). The next 30 teeth underwent all the above described group procedures but were treated with lower fluoride concentrations. Fluoride was extracted from enamel using 1 M KOH solution and analyzed using a fluoride ion-specific electrode. (3) Results: The findings revealed that slabs treated with NaF following calcium lactate pretreatment exhibited significantly greater enamel uptake of alkali-soluble fluoride compared to other substrates. This significant effect was not observed at lower fluoride concentrations. (4) Conclusion: The study demonstrates that pretreatment with calcium lactate followed by treatment with NaF at 226 ppm F significantly enhances the uptake of alkali-soluble fluoride in enamel compared to other fluoride types.

## 1. Introduction

Dental caries is a widespread and intricate global health problem that affects people of all ages and is shaped by a variety of etiopathological factors. These factors involve the balance between demineralization and remineralization, dietary habits and oral hygiene, the composition and flow of saliva, tooth structure, genetic predispositions, fluoride exposure, and various environmental and socioeconomic influences. The three categories of etiopathological factors contributing to enamel lesions that lead to dental caries are general, local, and iatrogenic factors [1]. General factors include genetic susceptibility, hormonal changes related to gender, aging, immune system factors, pregnancy-related changes, chronic illnesses, hormonal imbalances, vitamin levels, and socioeconomic conditions, all of which influence the risk of developing dental caries [2,3,4,5]. Furthermore, understanding and reducing caries risk requires analyzing local factors such as dental anatomy, oral hygiene practices, and the crucial function of saliva [6,7,8,9,10]. Additionally, issues like the premature loss of primary teeth, dental crowding, orthodontic treatment, dental restorations, and prosthetic work can lead to iatrogenic complications that impact oral health [11,12].

Enamel, dentin, cementum, and bone consist of insoluble calcium salts derived from carbonate, silicate, and phosphate ions. Calcium carbonate minerals can occur naturally in three distinct polymorphs, ranked by decreasing solubility: vaterite, aragonite, and calcite [13].

Teeth and bone are continually susceptible to demineralization, the primary process responsible for the loss of mineralized tissue throughout life [14,15], with a particular emphasis on teeth that sets them apart from bones, due to their anatomical arrangement and location [16,17]. Due to constant exposure to food, beverages, and oral microbiota, teeth have developed a remarkable resistance to localized demineralization, surpassing that of other mineralized tissues [18,19].

The demineralization process affects the enamel’s structure, starting with a superficial decalcification that renders the enamel surface porous and gives it a chalky appearance [20]. Decalcification occurs when calcium and phosphorus minerals are stripped from the tooth surface. When plaque remains on the enamel for extended periods, oral bacteria use sugars from the diet to produce acid. During an acidic attack, or a typical demineralization process, the chemical dissolution of both the organic and inorganic matrix components begins, causing the enamel to weaken and form white decalcification spots. Over time, these decalcified areas can progress to cavitation and cavity formation. This process is facilitated by the water content in enamel and dentin, which allows acid to diffuse into the tooth and minerals to diffuse out [21].

Fluorides are essential in preventing dental caries and are also used therapeutically to halt the progression of early carious lesions. The effectiveness of fluoride is primarily realized through topical application, which is further enhanced by good oral hygiene practices. Since the introduction of water fluoridation, the prevalence of dental caries has declined. However, the risk of dental fluorosis is primarily associated with systemic fluoride intake during the first six years of life [22].

Fluoride has a strong anti-cariogenic effect when applied topically through sources like fluoridated drinking water, toothpaste, mouthwash, and varnish. However, when fluoride is administered systemically through supplements such as drops, tablets, and lozenges, its impact on caries prevention is minimal [23].

The effects of different types and concentrations of fluoride on dental health have been extensively studied. Various fluoride compounds, such as sodium fluoride (NaF), amine fluoride (AmF), and monofluorophosphate (MFP), are commonly used in dental care products. NaF is effective in remineralizing enamel and preventing dental caries and therefore is commonly used in toothpaste and mouth rinses. It is often found in concentrations of around 1000–1500 ppm in toothpastes. MFP is a less reactive fluoride compound that is often combined with other fluorides to enhance its efficacy. It is frequently used in toothpaste formulations to provide a steady release of fluoride ions. AmF solution has surfactant properties that enhance its ability to adhere to dental surfaces, providing prolonged fluoride release and enhanced remineralization. AmF is commonly found in concentrations around 1400 ppm in toothpaste [24].

The concentration of fluoride in oral fluids is sustained by its release from bioavailable reservoirs in the teeth, oral mucosa, and, most crucially due to its link with the caries process, dental plaque. Oral fluoride reservoirs exhibit a dual nature, encompassing mineral reservoirs such as calcium fluoride or deposits contaminated with phosphate, as well as biological reservoirs, specifically fluoride bound to bacteria or bacterial fragments through calcium-fluoride interactions. Because of its ability to react with fluoride, calcium has been studied as a pretreatment to enhance intraoral fluoride retention after the use of a fluoride mouth rinse [25,26].

The regular use of a NaF mouth rinse with a concentrated formulation led to a considerable slowdown in the progression of lesions, whereas the fluoride solution with a lower pH exhibited a complete inhibition of lesion formation [27].

The pre-rinsing procedure involving calcium (Ca) before administering a fluoride (F) rinse at a concentration of 226 μg/g (ppm) has been known to stimulate the formation of deposits resembling calcium fluoride (CaF_2_) within human dental plaque, and the results demonstrated that the Ca pre-rinse induced large increases in plaque fluid and total plaque fluoride. However, without the Ca pre-rinse, 30% of the plaque F deposits were CaF_2_ or CaF_2_-like. Given that maintaining an elevated F concentration in the vicinity of a developing lesion may play an important role in the cariostatic effect of this ion, as well as the potential advantages of CaF_2_-like deposits as an F source, these results suggest that a Ca pre-rinse may increase the cariostatic effect of topical agents [28].

After fluoride treatments such as topical applications, varnishes, solutions, rinses, or dentifrices, salivary fluoride concentrations decrease exponentially in a biphasic manner to very low concentrations within a few hours. For treatments to be effective over periods longer than brushing and the following salivary clearance, fluoride needs to be deposited and slowly released. Calcium fluoride (or like) deposits behave this way because they are covered with a layer of phosphate and/or proteins, which makes CaF_2_ more soluble under in vivo conditions than in its pure form in inorganic solutions. Moreover, because of the phosphate groups on the surface of calcium fluoride globules, fluoride is believed to be released as the pH decreases, and the phosphate groups become protonated in dental plaque [29]. A precipitation reaction with calcium following fluoridation did not increase enamel fluoride uptake [30].

Research on enamel fluoride uptake affected by the site of application and type of fluoride concluded that enamel treated with the amine fluoride solution (AF) had significantly higher fluoride uptakes at all treated sites when compared to the NaF-treated specimens [31]. On the other hand, one study that compared the anti-caries efficacy of the two agents most widely used in fluoridated toothpastes, NaF and MFP, demonstrated that NaF was significantly more effective than MFP in preventing caries (*p* < 0.01) [32].

This study aimed to investigate if calcium pretreatment can proportionally increase fluoride reactivity with enamel related to different fluoride types at different concentrations.

**Null Hypothesis (H0):**  *Calcium pre-treatment does not significantly influence the enamel reactivity with different types of fluoride solutions.*

## 2. Materials and Methods

### 2.1. Type of Study

This parallel-group/treatment, blind, and randomized study was approved by the Ethics Committee of the School of Dental Medicine University of Zagreb under the protocol number (05-PA-30-17-4/2023).

All experimental procedures were conducted in accordance with the Declaration of Helsinki’s recommendations guiding physicians in biomedical research.

The total number of enamel slabs included in this study in the described procedures arranged for this research is 240—a number that includes 60 teeth (6 × 10 teeth × 4 slabs) cut into 240 samples of enamel slabs. The sample size was calculated based on a pilot study with 4 samples/group and our ability to detect differences between the groups and treatments. It was checked for all 4 substrates/conditions with power analysis using Stata SE 16.1 for Mac (StataCorp LLC, College Station, TX, USA). Using 8 samples/group/condition would allow us to detect differences at a significance level of 5% with 80% power. The total number of enamel slabs planned for this study is 240.

### 2.2. Enamel Slab Preparation

A total of 60 non-carious human wisdom teeth were used, according to the inclusion criteria as follows: non-carious wisdom human teeth extracted for orthodontic reasons; donor’s age ranged from 21 to 30; intact enamel surface; suitable for cutting into four equal sections; clear and identifiable enamel surfaces for measurement purposes.

Each tooth was cut into 4 enamel slabs marked with a number and letter (a, b, c, d), covered with laboratory wax with inserted orthodontic wire for easier handling and marking flags, and randomly allocated into one of the treatment groups, as follows.

The first 30 teeth, cut and allocated to four groups according to letter, were treated for 5 min as follows:

Group A—slabs pretreated with solution of calcium lactate (150 mM) followed by one of 3 different fluoride solutions (NaF, MFP, AmF; the characteristics of these products are presented in Table 1): (1–10) A slabs—NaF (226 ppm free fluoride ion); (11–20) A slabs—MFP (22 ppm free fluoride ion); (21–30) A slabs—AmF (226 ppm free F^−^ ion).

Group B—slabs in “fluoride only” group were treated with 3 different fluoride solutions, as follows: (1–10) B slabs—NaF (226 ppm free fluoride ion); (11–20) B slabs—MFP (226 ppm free fluoride ion); (21–30) B slabs—AmF (226 ppm free fluoride ion). 

Group C—slabs in the “calcium only” group: all 30 C slabs were treated with 150 mM calcium lactate solution. 

Group D (negative control group)—all 30 D slabs were treated with deionized water. The next 30 teeth underwent all of the above-described group procedures but were treated in the described protocol with NaF 70 ppm, MFP 65 ppm, and AmF 75 ppm; all ppm of free F ion concentration of all three types of fluorides.

### 2.3. Extraction of Alkali-Soluble Fluoride

After the treatments, alkali-soluble fluoride extraction was conducted by immersing the enamel slabs in a 1 M KOH solution (0.25 mL per sample) for 24 h under agitation (160 rpm) at room temperature, using the method outlined by Caslavska [33]. After the 24 h period, all extracts were buffered with 0.5 mL of TISAB III solution containing 1.0 M HCl, and the samples were carefully removed from the tubes. The extracted solutions were then subjected to analysis using an ion-specific electrode (DRZAC ELEKTRODE SP ROSIN GRGET) connected to an ion analyzer (Expandable ionAnalyzer EA 940 Orion Research, MA, USA) that had been previously calibrated using fluoride standards prepared to match the sam-ples. The concentration of alkali-soluble fluoride was expressed as the amount per surface area of the enamel slabs (μg F/cm2).

### 2.4. Statistical Analyses

The collected data were entered, inputted, and arranged in a Microsoft Office Excel file. The normality of data distribution was checked, and alkali-soluble fluoride data were transformed using log10. The Wilcoxon Matched Pairs Test was used for comparisons among the treatment groups of the three different fluoride type solutions, and Friedman ANOVA was employed to analyze the impact of both the substrate and treatments. The study design is schematically presented in Table 2.

## 3. Results

All fluoride treatments, pretreated or not with calcium, significantly increased the concentration of alkali-soluble fluoride in the enamel samples when compared with the negative control group (*p* < 0.05). A significantly greater enamel uptake of the alkali-soluble fluoride was measured in group NaF 226 ppm F pretreated with a calcium lactate solution when compared to other tested samples, including calcium lactate or NaF alone (3.919 vs. 0.519 mcg F/cm^2^, *p* < 0.05). No significant difference in the enamel uptake of KOH-soluble fluoride was observed among calcium lactate and the different concentrations and types of fluoride substrates AmF or MFP (0.897 vs. 0.479 mcg F/cm^2^ and 0.571 vs. 0.561 mcg F/cm^2^, respectively, *p* > 0.05).

The results suggest that both concentrations of NaF have distinctive effects on the enamel uptake of KOH-soluble fluoride, but NaF 226 ppm F show a broader range of significant differences. In this case, pretreatment with calcium lactate followed by NaF showed significant differences compared to fluoride only treatment at a higher concentration of 226 ppm of F^−^ ions (*p* < 0.05). However, at the lower concentration of 70 ppm, there was no significant difference in fluoride uptake between the same groups (*p* > 0.05).

**Figure 1:**
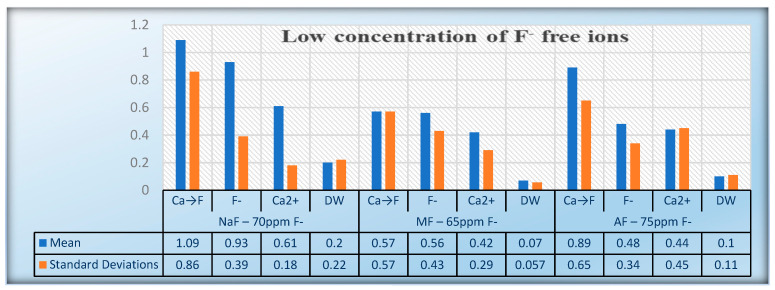
Uptake of alkali soluble fluoride during treatment with low concentration of F free ions.

**Figure 2:**
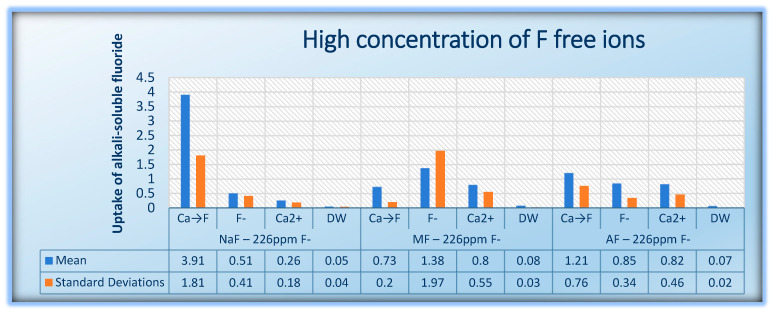
Uptake of alkali soluble fluoride during treatment with high concentration of F free ions.

The descriptive statistics considering 95% CI are presented in Table 3. The mean value and standard deviation (SD) of alkali-soluble fluoride after pretreatment with calcium lactate solution or/and treatment with different concentrations and types of fluoride solutions of enamel slabs are presented in Table 3 and Table 4 and in Figure 1 and Figure 2.

## 4. Discussion

The bacterium *Streptococcus mutans*, as based on evidence, is a primary factor in the initiation of caries, while bacteria of the genus *Lactobacillus* play a significant role in the progression of caries, especially in dentin. Other bacteria can also contribute to caries, including species from the *mitis*, *anginosus*, and *salivarius* groups of streptococci, *Enterococcus faecalis*, *Actinomyces naeslundii*, *A. viscosus*, *Rothia dentocariosa*, *Propionibacterium*, *Prevotella*, *Veillonella*, *Bifidobacterium*, and *Scardovia* [34].

Fluoride has emerged as the leading factor contributing to the decline in caries prevalence [35]. The primary mechanism by which fluoride influences the progression of dental caries is through its impact on the de- and remineralization processes that take place in dental hard tissues. When fluoride is present in the plaque fluid during acid production by bacteria, it can penetrate the subsurface along with the acids, adsorb to the crystal surface, and provide protection against dissolution of the crystals [36]. Furthermore, it has been suggested that fluoride ions may have an impact on the physiology of microbial cells, including cariogenic streptococci, leading to indirect effects on the process of demineralization. This suggests that fluoride’s action on dental caries is not only limited to its direct effects on the mineralized tissues but also involves interactions with the microbial ecology of the oral cavity [37,38,39,40,41].

After using a fluoridated dentifrice, plaque fluoride concentrations can remain elevated for several hours [42,43].

Fluoride has a strong affinity to both the organic and inorganic components of plaque and can be found in ionic, ionic, and strongly bound forms. Although the amount of fluoride in the ionic fraction is considerably larger than in the ionic pool, it adds to the amount of ionic fluoride in plaque fluid, which is responsible for the cariostatic action of fluoride [44].

This study investigated if fluoride reactivity with tooth substrates can be enhanced by a complex measure: calcium pretreatment of the enamel surface followed or not by three different fluoride types (NaF, MF, AF) with two different concentrations (avg. 70 ppm and 226 ppm free F^−^ ions). Although higher concentrations, such as those used in professional fluoride treatments (e.g., gels and varnishes with around 10,000 ppm F) are more effective in treating and preventing dental caries, everyday products designed for regular use to maintain oral health, like toothpaste and mouth rinses, contain lower fluoride concentrations (1000–1500 ppm F) to minimize the risk of fluorosis [45]. The present research highlights the importance of fluoride concentration and the method of application.

This study demonstrated that all fluoride treatments, whether pretreated with calcium or not, significantly increased the concentration of alkali-soluble fluoride in enamel substrates compared to the negative control group (*p* < 0.05). These results are consistent with previous research [46] showing that fluoride application enhances enamel fluoride content, which is crucial for caries prevention.

The advantage of NaF solution in KOH-soluble fluoride uptake compared with AmF or MFP solutions, with calcium lactate pretreatment of enamel followed by different concentrations and types of these fluoride solutions, is supported by research from Ekambaram M (2010) [9,32], with differing trends. They found that 500 ppm NaF dentifrice resulted in the remineralization of carious lesions by virtue of a significant decrease in lesion depth; whereas dentifrices that contained AmF and MFP decelerated the progression of demineralization. Some other studies show significant differences between NaF compared to MFP in preventing caries effects [47].

The tested fluoride concentrations of 70 ppm and 226 ppm F are significantly lower than the typical levels used for enhancing fluoride reactivity with tooth structures, such as those found in fluoride gels and varnishes, which usually contain around or over 10,000 ppm F. This specific concentration was chosen based on prior studies demonstrating that, when applied after a calcium rinse, a 226 ppm NaF solution can boost fluoride levels in saliva and dental biofilm by 5 to over 20 times [48,49,50], as well as to minimize fluoride side effects in an attempt to successfully reduce dental fluorosis without compromising caries prevention [51]. In our study, an even lower concentration of NaF was tested (70 ppm), but the boosting effect of calcium lactate was not shown. There might be a limitation of the calcium lactate effect related to the concentration of available free fluoride ions. Similarly, the concentration of free fluoride ions may explain significantly a lower boosting effect for MFP and AmF. It is well known that each of these two types of fluorides needs other steps to release free ions from their compounds: MFP needs bacterial hydrolytic degradation [52], and AmF needs interaction with salivary mucins and proteins [53]. This was not covered in our study protocol. Adding artificial saliva or human saliva to enamel treatments can enhance the release of free fluoride ions from MFP and AmF and improve fluoride uptake by enamel. Both artificial and human saliva mimic the natural oral environment, which is crucial for studying fluoride dynamics. They help maintain a constant pH and provide a source of calcium and phosphate ions, which are essential for the remineralization process. Human saliva contains enzymes and ions that can help break down fluoride compounds, enhancing the release of free fluoride ions from MFP and AmF. This increased release can lead to more available fluoride for enamel uptake. The presence of calcium and phosphate ions in saliva can promote the formation of fluorapatite on the enamel surface, which is more resistant to acid attacks compared to hydroxyapatite. This process enhances the overall fluoride uptake by the enamel [54,55]. Our study indirectly confirms that calcium lactate pretreatment followed by low-dose MFP or AmF fluoride treatment cannot enhance fluoride uptake by the enamel per se, probably due to lack of bacterial and saliva functions.

In a clinical setting, these rinses are intended for daily use rather than a one-time application. The recommended rinsing time is 1 min [56,57]; therefore, in our study we used a 5 min reaction time for each solution. This extended duration was chosen to enhance reaction sensitivity, enabling the detection of subtle differences among groups.

Calcium lactate has been shown to have a beneficial effect on the release of fluoride ions from both MFP and AmF, particularly in the presence of saliva. The interaction between calcium lactate and fluoride compounds can enhance the bioavailability and retention of fluoride on the enamel surface, thereby promoting better remineralization and increased fluoride uptake [58]. Similarly, calcium ions play a crucial role in stabilizing fluoride on the enamel surface, especially when used with AmF. AmF tends to form a more homogenous and less soluble layer on the enamel, which is further stabilized by the presence of calcium ions, thereby improving the overall efficacy of fluoride treatments in caries prevention [59].

One limitation of this study might be the conduction in a controlled laboratory setting, which may not fully replicate the complexity of the oral environment. Real-life conditions, such as the presence of other minerals, interactions with saliva, and exposure to various dietary factors, could influence the outcomes differently than observed in the controlled experimental setup. The study explored fluoride uptake at two specific concentrations (226 ppm and lower concentrations of 70 ppm, 65 ppm, and 75 ppm for different fluoride types). Although this range sheds light on the effects of varying fluoride levels, it does not cover the full spectrum of fluoride concentrations that might be found in various dental products, which could limit the applicability of the findings across all clinical scenarios. This research focused exclusively on calcium lactate as a pretreatment agent, without comparing it to other calcium compounds that might have differing impacts on fluoride uptake and enamel remineralization. Additionally, the study lacked a long-term follow-up to evaluate the persistence of fluoride uptake and its effectiveness in caries prevention over time. As a result, it remains uncertain whether the increased fluoride uptake observed in the short term will lead to prolonged enamel protection.

A limitation of the present study is also the lack of comparison with other advanced techniques that measure fluoride concentration in tissue. While this study utilized Ion-Selective Electrode (ISE) analysis to measure fluoride uptake in enamel, these methods may not provide the same level of sensitivity, spatial resolution, or detailed fluoride distribution analysis as other available techniques. Future research should consider incorporating or comparing these findings with methods such as Micro-PIXE (Proton-Induced X-ray Emission), Electron Probe Microanalysis (EPMA), Nuclear Magnetic Resonance (NMR), or Secondary Ion Mass Spectrometry (SIMS), which may offer a more comprehensive understanding of fluoride uptake and localization within enamel tissues. Micro-PIXE (Proton-Induced X-ray Emission) and Electron Probe Microanalysis (EPMA) provide spatially resolved measurements of fluoride content at a microscopic level, which can reveal heterogeneity in fluoride distribution within the enamel. These techniques, while more complex and resource-intensive, offer a detailed view of fluoride localization that may not be achievable with the methods used in this study [60,61]. Furthermore, Nuclear Magnetic Resonance (NMR) and Secondary Ion Mass Spectrometry (SIMS) allow for non-destructive analysis and the potential to study fluoride binding in the organic and inorganic components of the enamel. These methods could provide complementary data, enhancing our understanding of the fluoride–enamel interaction [62,63]. The demineralization processes did not include bacterial effects in our study, so the caries process in vivo may differ from our setup.

The findings of this study open several promising avenues for future research and clinical application. Firstly, the significant enhancement of fluoride uptake by enamel following calcium lactate pretreatment, particularly with sodium fluoride at a concentration of 226 ppm, suggests a potential to refine existing fluoride treatment protocols. Future studies should explore the long-term effects of this combination to determine whether the increased fluoride uptake translates into sustained enamel protection and reduced caries incidence over time. Further research could explore a broader range of fluoride concentrations, particularly in everyday dental products like toothpaste and mouth rinses, to assess whether the benefits observed at 226 ppm NaF can be replicated or even improved at different levels.

Additionally, investigating the impact of other calcium compounds as pretreatment agents could provide insights into alternative methods for enhancing fluoride reactivity with enamel. Such studies could compare calcium lactate with other calcium salts to identify the most effective agent for maximizing fluoride uptake.

## 5. Conclusions

The findings of this study highlight the synergistic effect of calcium lactate pretreatment in enhancing enamel fluoride uptake when followed by sodium fluoride in a concentration of 226 ppm free F ions but not in lower F concentrations. Calcium lactate pretreatment enhances sodium fluoride enamel uptake but has little to no effect on other types of fluorides, like amine or monofluorphosphate fluoride. This combination of calcium lactate and sodium fluoride shows significant potential in clinical applications aimed at maximizing fluoride incorporation into the enamel and minimizing the possibility of fluorosis development. Future studies should continue to explore the interactions between calcium and fluoride treatments to optimize dental care protocols.

## Figures and Tables

**Table 1:** Fluoride type solution and calcium lactate solution characteristics used in this study.

Treatment Substance	Composition	Manufacturer	OT No.
Calcium lactate pentahydrate 150 mM	Calcium lactate pentahydrate—4.624 g Purifying water—100 mL	Farmalabor, GLJZ—Croatia	R2220462, 161022
Sodium fluoride 500 ppm (226 ppm free F ion)	Sodium fluoride—0.05 g Purifying water—100 ml	Farmalabor, GLJZ—Croatia	R2119627, 161022
Sodium fluoride 155 ppm (70 ppm free F ion)	Sodium fluoride—0.0155 g Purifying water—100 mL	Farmalabor, Hamapharma—Croatia	R2119627, 0070922
Sodium Monofluorophosphate 500 ppm (65 ppm free F ion)	Sodium Monofluorophosphate—0.05 g Purifying water—100 mL	NEVIA (Magdis d.o.o), Hamapharma—Croatia	100084372120200821, 0070922
Sodium Monofluorophosphate 1720 ppm (226 ppm free F ion)	Sodium Monofluorophosphate—0.1720 g Purifying water—100 mL	NEVIA (Magdis d.o.o), Hamapharma—Croatia	100084372120200821, 0070922
Aminofluoride 500 ppm (75 ppm free F ion)	Aminofluoride— 0.05 g Purifying water—100 mL	Fagron, Hamapharma—Croatia	104/22, 0070922
Aminofluoride 1500 ppm (226 ppm free F ion)	Aminofluoride— 0.15 g Purifying water—100 mL	Fagron, Hamapharma—Croatia	104/22, 0070922

**Table 2:** Schematic representation of the study design.

Group A			Group B			Group C	Group D
NaF—a *n* = 10	MFP—a *n* = 10	AmF—a *n* = 10	NaF—b *n* = 10	MFP—b *n* = 10	AF—b *n* = 10	Ca *n* = 30	Deionized Water *n* = 30
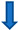	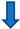	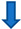	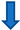	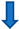	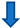	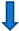	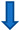
Calcium lactate pretreated for 5 min followed by 3 different fluoride solutions: (1–10) A NaF, (11–20) A-MFP, (21–30) A—AmF			Treated for 5 min with 3 different fluoride solutions as follows: (1–10) B- NaF, (11–20) B-MFP, (21–30) B—AmF			Treated with 150 mM calcium lactate solution	Treated with deionized water (negative control)
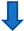			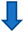			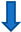	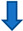
Fluoride extraction was conducted by immersing the enamel slabs in a 1 M KOH solution for 24 h under agitation at room temperature. After the 24 h period, all extracts were buffered with TISAB III solution containing 1.0 M HCl							
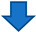							
The extracts were analyzed using a fluoride ion-specific electrode							

**Table 3:** The medians and percentiles of fluoride uptake (226 ppm free F ions).

226 ppm Free F^−^ Ion	NaF				MFP				AmF			
	Ca→F	F^−^	Ca^2+^	DW	Ca→F	F^−^	Ca^2+^	DW	Ca→F	F	Ca^2+^	DW
**Median**	3.317	0.391	0.196	0.041	0.673	0.565	0.585	0.078	0.866	0.762	0.671	0.071
**Percentiles** **25%/75%**	2.736 / 5.452	0.247 / 0.524	0.157 / 0.294	0.024 / 0.058	0.612 / 0.873	0.495 / 1.282	0.502 / 0.742	0.048 / 0.11	0.735 / 1.244	0.608 / 1.02	0.534 / 0.882	0.057 / 0.088

**Table 4:** The medians and percentiles of fluoride uptake (avg. 70 ppm free F ions).

	NaF–70 ppm F^−^				MFP–65 ppm F^−^				AmF–75 ppm F^−^			
	Ca→F	F^−^	Ca^2+^	DW	Ca→F	F^−^	Ca^2+^	DW	Ca→F	F	Ca^2+^	DW
**Median**	0.684	0.866	0.591	0.113	0.351	0.382	0.355	0.053	0.862	0.382	0.222	0.068
**Percentiles** **25%/75%**	0.592 / 1.133	0.729 / 0.985	0.500 / 0.737	0.075 / 0.211	0.184 / 0.731	0.216 / 0.833	0.162 / 0.597	0.041 / 0.098	0.227 / 1.205	0.252 / 0.675	0.111 / 0.49	0.033 / 0.158

## Data Availability

The original contributions presented in the study are included in the article; further inquiries can be directed to the corresponding authors.
